# The More Hopeful Aspect of Intra-Cranial Tumours

**Published:** 1907-09-14

**Authors:** 


### The More Hopeful Aspect of Intra-cranial Tumours.

The conclusion that any individual patient is the subject of an intra-cranial tumour is manifestly a diagnosis of an extremely serious order. Even where there is reason to believe that the growth is syphilitic in nature, and is susceptible, therefore, to the influence of appropriate medicinal remedies, the outlook is not free from anxiety. Though a degree, and even a considerable degree, of improvement may be so obtained, there are often associated cerebral changes the consequences of which do not disappear as do the more direct evidences of the neoplasm. And there is good reason to believe that a gumma itself, when it has undergone a more or less complete caseation, is to a corresponding degree uninfluenced by anti-syphilitic medication. Hence, even a diagnosis that a given intra-cranial tumour is an expression of syphilis, carries the anxiety that permanent ill-effects, bodily or mental or both, may be the consequence. Still more alarming is the general outlook when syphilis cannot be advanced as an explanation. Here the hope of checking the extension of the growth, and still more of removing the growth by the use of medicines must be admitted to be at a minimum. Further, it must be allowed that the high expectations raised some years ago, and more particularly by the results of such workers as Sir Wm. Macewen and Sir Victor Horsley, that surgery would prove competent to deal successfully with tumours within the skull, have not been realised in practice. It is now recognised that only a small proportion of such tumours can be treated in this fashion. Possibly surgery may yet have greater triumphs in this direction, but at the present date the position is as just stated. The general proposition, therefore, remains true, that a diagnosis of intra-cranial tumour involves a prognosis framed in terms of great anxiety and gravity. There are, however, certain facts which, at least in some measure, help to relieve the outlook, and it is important that these, even though of only occasional or exceptional occurrence, should be borne in mind, equally with those of more forbidding aspect.

The more hopeful view of the clinical possibilities attached to a diagnosis of intra-cranial tumour is afforded by certain cases which show that, sometimes at all events, such tumours pass into a condition, and even into a permanent condition, of quiescence. There are post-mortem records entirely beyond dispute in which a shrivelled or atrophied tumour has been found in some part of the brain when the personal history of the patient included the history of an illness, which occurred many years before death, and which, manifestly, was the result of some gross intra-cranial disease. Here the position is little removed from that of direct demonstration. And the conclusion is obvious, not only that an intra-cranial tumour may cease to extend, or may, indeed, actually shrink, but that the presence of an inert mass within the brain is compatible with many years of life undisturbed by the presence of symptoms of cerebral disease. The inference seems to be justified that it is not so much the presence as the active extension of a tumour within the skull that gives rise to the classical symptoms of intra-cranial neoplasm. This conclusion is also supported by those occasional experiences in which a post-mortem examination detects the presence of a cerebral tumour which had not given rise to any symptoms during life, and the existence of which was entirely unsuspected. It is presumably, therefore, less the mechanical effects of a tumour-mass, than the irritation and congestion associated with its active growth, that are the directly responsible causes for the clinical disturbances which indicate the diagnosis of intra-cranial tumour. If such active growth ceases, and it certainly sometimes does so, the disturbances dependent on it may come to an end. This, it is true, does not exclude the possibility of renewed activity. But the favourable chance, small as it is, may well be welcomed in circumstances which are generally held to banish even the smallest ray of hope.

There are still other facts which help somewhat to relieve the outlook in a diagnosis of intra-cranial tumour. These are provided by cases in which, after a serious illness characterised by symptoms of obviously cerebral origin, the patient, generally a child or adolescent, makes an unexpected recovery. For the most part such recovery is qualified by the profound misfortune of blindness, due, as is found on examination, to post-neuritic optic atrophy. There can be little doubt that these are cases of tumour; and of obvious importance in relation to them are the instances already mentioned in which autopsy has revealed a shrunken tumour-mass many years after an illness distinguished by symptoms of cerebral origin. Thus it may be positively stated that in a certain number of instances intra-cranial tumours cease to grow; that such cessation may be attended with the disappearance of all active symptoms; and that in many of these cases there is blindness as a result of optic atrophy secondary to neuritis. These facts lend encouragement to treatment, and especially to the proposal to trephine the skull, even when removal of the tumour is out of the question. This measure will frequently cause optic neuritis to subside, and may thus save the patient's sight. When it is also realised that, at least in an occasional case, the tumour may cease to grow, there is an urgent claim to avoid an attitude of helpless despair.

